# Fetal origins of obesity: a novel pathway of regulating appetite neurons in the hypothalamus of growth-restricted rat offspring

**DOI:** 10.1007/s00404-023-07108-3

**Published:** 2023-06-28

**Authors:** Weiling Han, Zhaoyi Song, Dan Shan, Qingyun Shi

**Affiliations:** 1 Department of Obstetrics, Beijing Obstetrics and Gynecology Hospital, Capital Medical University, Beijing Maternal and Child Health Care Hospital, No. 251 Yao Jia Yuan Road, Chao Yang District, Beijing, 100026 China; 2 STI-Zhilian Research Institute for Innovation and Digital Health, Beijing, China; 3 Department of Obstetrics and Gynecology, The People’s Hospital of Yongcheng, Dongcheng District, Yongcheng City, Henan Province China

**Keywords:** Fetal growth restriction, DNMT1, Bsx, NPY, Hypothalamus

## Abstract

**Purpose:**

Fetal growth restriction causes a series of sequelae, some of which, such as hyperphagia, reduced satiety and postnatal obesity, are believed to be associated with embryonic hypothalamic neurons impairment. The mechanisms underlying the linkage of fetal brain injuries to break the energy homeostasis have not been elucidated completely. Here, we aim to investigate the effect of intrauterine energy restriction on remodeling appetite neurons in the hypothalamus of fetal and postnatal infant rats.

**Methods:**

Low-protein (8%) diet combined with 75% energy restriction was used to establish an animal model. Rats offspring brain tissues, harvested from embryo day 18 and postnatal infant day 1, were sampled for dependent regulator analyses and master neuron assessment.

**Results:**

Growth-restricted rats showed the increased expression of Bsx and NPY in the hypothalamus as well as remodeling hypothalamic neurons differentiation compared to controls. Intriguingly, in cells cultured in vitro test, we found that activated effects of Bsx and NPY could be exacerbated by DNMT1 inhibitor.

**Conclusions:**

In embryonic and early postnatal stage of FGR rats, we detected high concentrations of orexigenic neurons in the hypothalamus. DNMT1 activity is correlated with early embryonic neurogenesis by mediating the expression of Bsx and NPY. It may be one of the reasons for the abnormal development of the appetite regulation pathway and higher susceptibility to obesity in FGR offspring.

## What does this study add to the clinical work

**Table:** 

In embryonic and early postnatal stage of FGR rats, we detected high concentrations of orexigenic neurons in the hypothalamus.	
DNMT1-mediated methylation levels in the Bsx promoter region were associated with dysfunctional differentiation of hypothalamic NPCs.	
The findings suggested that the changes of early undernutrition on offspring DNA methylation preset and programmed obesity and metabolic syndrome.	

## Introduction

Fetal growth restriction (FGR), defined as an estimated fetal weight < 10th percentile for gestational age [1], is mainly caused by dysfunction of the fetal–placental perfusion, resulting in hypoxia and acidosis in the fetal circulation [2]. Except for higher rates of fetal and neonatal morbidity and mortality, the damage can even last until adulthood, leading to an increased risk of obesity and metabolic diseases, also known as developmental origins of health and disease (DoHaD) [3–6]. Thereby, such chronic diseases of adulthood have been considered as having early developmental origins in the perinatal period.

Compelling evidences show that the early-stage programming in hypothalamus development, in interaction with other epigenetic marks, adds powerful layers of susceptibility to metabolic diseases [7, 8]. During embryonic development, hypothalamic neural precursor cells (NPCs) in hypothalamus can differentiate into two types of appetite-regulating neurons, Neuropeptides Y (NPY) and proopiomelanocortin (POMC). NPY stimulates food intake and reduces energy consumption, while POMC inhibits food intake and promotes energy consumption. The dynamic balance with antagonistic functions maintains energy homeostasis [9, 10]. Moreover, infants small for gestational age demonstrate programmed dysfunction of NPCs proliferation, which preferentially differentiate to NPY as compared to POMC neurons [3]. To study obesity intensively, it is crucial to understand cellular and molecular mechanisms of NPCs differentiation. In close relationship with hyperphagia and locomotory behavior, Brain-Specific Homeobox (Bsx) was reported as a required factor in the transcriptional network of NPY/AgRP expression [11]. However, the specific molecular linkage of Bsx and NPY in FGR offspring is unclear.

DNA methylation, one of the main kinds of epigenetic modifications, has been thought to be an important mediator for neuron differentiation regulating [12–15]. DNA methylation is the covalent addition of methyl group catalyzed by DNA Methyltransferase (DNMTs) [16], leading to gene silence or expression down-regulation. The members of DNMTs family contain DNMT1, DNMT3a / 3b, and DNMT3-l [17]. Reports have evidenced that in small for gestational age offspring, impaired neurogenesis is mediated via reduction in DNMT1 expression partly resulting in NPCs differentiation [18], suggesting that DNMT1 may be an potential target factor in altered neurogenesis for appetite regulation of FGR offspring.

Overall, we hypothesized that DNMT1, Bsx and NPY might be involved in triggering dysfunctional differentiation of hypothalamic NPCs in FGR rat offspring, which directly led to the increased susceptibility to obesity in adulthood. Using both animal modeling and in vitro hypothalamic NPCs culturing, our study explored the possibility of a mechanistic link between DNMT1, Bsx and NPY pathways. This research will help to provide a theoretical basis and clinical guidance for the early interventions of FGR, reducing the risk of adverse effects and improving the quality of life in the long term.

## Materials and methods

### Animal modeling and grouping

Low protein content (8%) feeding combined with 75% maternal food restriction was used to establish an FGR rat model [19]. There were 24 first-time pregnant Sprague–Dawley (SD) rats (*N* = 24) (Beijing Vital River Laboratory Animal Technology Co., Ltd., Beijing, China) weighing approximately 230 g and aging 90–120 days. They were housed in a specific pathogen-free (SPF) animal facility with a sterile barrier system, constant temperature (20 °C), relative humidity of 60–70%, and a 12-h light/dark cycle. Water was not restricted during the whole gestation. On the first day of gestation (the first day of suppository was recorded as the first day of gestation), they were randomly divided into two groups. The FGR group (*N* = 12) rats were fed 8% protein feeding combined with 75% maternal food restriction. The control group rats (*N* = 12) were provided with breeding feed with no energy restriction. The pregnant rats in the control and FGR groups were randomly divided into two cohorts, the embryonic day 18 (E18) cohort, which was cesarean delivered at E18 (*N* = 6 pregnant rats), and the postnatal day 1 (P1) cohort, which was natural delivered (*N* = 6 pregnant rats). All of the offspring were weighed and recorded immediately delivered, whether cesarean or natural delivery. Two standard deviations lower than the average weight of control group offspring were considered successfully modeling. As fetal gender may confuse the results, only female rat offspring were used in this study. Females were distinguished from males by the presence of a uterus duplex.

### Hypothalamic NPCs cultural in vitro

The hypothalamus of female rat offspring was used in this study. Fetuses were taken out and placed at pre-chilled PBS. After being weighed and recorded, twelve female rat offspring were collected from the FGR group and the control group (*n* = 6 in each group) for hypothalamic NPCs in vitro culture. The hypothalamus was bluntly dissected under aseptic conditions. Then, meninges and blood vessels were removed. They were cut into small pieces and digested with 0.1% trypsin–EDTA and 0.01% DNase for 30 min in a 37℃ carbon dioxide incubator. Trypsin was inactivated by fetal bovine serum (FBS). Then, the samples were filtered and centrifuged at 1500 rpm for 5 min. After discarding the supernatant, the samples were resuspended in serum-free complete medium (5 × 10^4^ cells/ml) (the complete medium consists of neurobasal™ medium, 1% penicillin–streptomycin mixture, 2% B-27 additive, 20 ng/mlFGF2, 20 ng/ml EGF, 1 μg/ml heparin and 2.5 μg/ml L-glutamine). Then, the cells were cultured in a 37 ℃ carbon dioxide incubator for eight days. The medium and FGF2 (10 ng/ml) were exchanged every three days. On the 8th day, the neurosphere cells were recorded as S0. S0 cells were collected and digested with 0.1% trypsin–EDTA at 37 ℃ for 5 min to dissociate them into single cells. Then, the samples were filtered and centrifuged at 1000 rpm for 5 min. After discarding the supernatant, they were re-inoculated into a complete culture medium (5 × 10^4^ cells /ml) and cultured for eight days according to the above culture conditions. The cells were recalled as S1. S1 cells were collected for induction and differentiation culture. They were dissociated into single cells, filtered, and centrifuged according to the above method. Then, They were re-inoculated into a differentiation medium (neurobasal™ medium, 1% penicillin–streptomycin mixture, 2% B-27 additive, 2.5 μG/MLL glutamine, without FGF2, EGF and heparin). The S1 cells in each group were randomly divided into two subgroups. One added DNMT1-specific inhibitor, 5-AZA (GIPBIO, 20 μM), into the culture medium; the other did not add inhibitors. Both were cultured in a 37 ℃ carbon dioxide incubator for eight days. Then, the cells were collected for subsequent experimental detection.

Maternal rats in the P1 cohort were fed until natural delivery. Six brains of female rat offspring were collected (*n* = 6 in each group) for hypothalamic NPCs in vitro culture. The process was the same as mentioned above.

### Immunofluorescence

Paraffin sections were used for IF. After dewaxing, antigen repairing, and detection area delineating, samples were permeated with 0.3% Triton X-100. Then, they were sealed and incubated in a wet box at room temperature for two hours. Next, NPY antibody (dilution ratio 1:500) was added. Fluorescent labeled Goat-anti-Rabbit antibody was used as a secondary antibody (dilution ratio 1:200). DAPI (dilution ratio 1:50) was used for nuclei counterstain. The sealing slices shall be dried in a 60 ℃ oven and sealed with anti-fluorescence quenching sealing agent. The images were collected by inverted fluorescence microscope (Nikon Ci-S, Nikon DS-U3) in a 100 × field of vision. Image J software was used for semi-quantitative analysis of fluorescence values.

### RT-PCR

RNA was extracted from hypothalamic tissue and neurosphere cells in this section. cDNA was obtained through reverse transcription reaction. Dilute it ten times for amplification by real-time quantitative PCR (RT-qPCR). The 2^− ΔΔ CT^ method (livak method) was used for quantitative analysis. Primer and internal reference sequence were as follows:

GAPDH: F: 5′- GGCAAGTTCAACGGCACAG-3′

R: 5′- CGCCAGTAGACTCCACGACA-3′

DNMT1: F: 5′- CACTGTTCCTCCTTCTGCCATCAAT-3′

R: 5′- TCATCGTCCTTAGCGTCGTCGTA-3′

NPY: F: 5′- GCTCTGCGACACTACATCAATCTCAT-3′

R: 5′- GCAAGTTTCATTTCCCATCACCACAT-3′

Bsx: F: 5′- CGGTGCCTTCGTGCTTACAGAG-3′

R: 5′- GCCGCAGGAACAGTCTAGAGTCT-3′

### Western blot

Total protein was extracted from hypothalamic tissues. The target protein was separated and purified by SDS–polyacrylamide gel electrophoresis. 10 μl of protein samples were added to each hole. Constant voltage power was supplied for electrophoresis (concentrated gel 80 V, separation gel 120 V). Electrophoretic separation would stop when the target protein was located at the best resolution position 1/3 below the surface of the separation gel. Then, samples were transferred to a PVDF membrane by constant current (200 mA) for 90 min at 4 ℃ (0.45 μm thickness of PVDF). Next, they were sealed for 2 h at room temperature with the sealing liquid containing 5% skimmed milk powder (prepared with 0.5% TBST). Then, the primary antibody was added after dilution (DNMT1 1:1000, Bsx 1:2000, NPY 1:1000). HRP-labeled Goat-anti-Rabbit antibody was used as a second antibody (1:5000 dilution). ECL kit was used for color exposure, and then gel images were collected by an automatic chemiluminescence image analysis system. Image J software was used for grayscale value analysis.

### Methylation-specific PCR

Hypothalamic tissues and neurosphere cells were used as samples. DNA extraction shall be carried out according to the operating instructions of the DNA extraction kit. Then the DNA samples were transformed into bisulfite by PCR-cycler and were purified. Then, DNA samples were amplified by methylation-specific PCR. The samples were concentrated through DNA agarose gel electrophoresis with 140 V direct current for 30 min. The loading quantity of the sample on each pore was 5 μl. Primer sequences were as follows (M-Bsx: Bsx methylated primer pair; U-Bsx: Bsx unmethylated primer pair):

M-Bsx: F: TTCGTAGTTTTTTTATTGGTTTTTC

R: AAACCTACACCTACTCCTAATCGTC

U-Bsx: F: TGTAGTTTTTTTATTGGTTTTTTGG

R: AAACCTACACCTACTCCTAATCATC

### Data analysis

Mean and standard deviation (SD) were used to describe the average weight of rats. Differences between the FGR and control group were analyzed by ANOVA with Dunnett’s post hoc test. The relative expression levels of mRNA were normalized to GAPDH. The data were statistically analyzed by spss23.0 software. *P* < 0.05 was considered statistically significant.

## Results

### The average birth weight in the FGR group was significantly lower than in the control group

The birth weight of rat offspring we used in the subsequent analyses in the FGR group was significantly lower than that in the control group (*P* < 0.05) (Table 1). The birth weight in the FGR group met the diagnosis criteria of FGR as it was lower than the average birth weight of the control group by 2 SD.

**Table 1:** Comparison of birth weight between FGR and control group ( $\bar{x}$± s, g)

	FGR	Control	*P*
	*n* = 12	*n* = 12	
E18	1.28 ± 0.05	1.72 ± 0.07	< 0.001
P1	5.22 ± 0.21	7.48 ± 0.22	< 0.001

E18: Embryonic day 18, P1: Postnatal day 1, *FGR* fetal growth restriction

### The expression of NPY in the hypothalamus of FGR offspring increased

In rats, the peak of neurogenesis is on gestational days 10.5–16.5. NPY can be detected for the first time on day E14.5. Embryo day 18 and postnatal day 1 rat offspring were chosen as study subjects to confirm the expression difference of NPY and consistency of the difference in or out-utero.

**Fig. 1:**
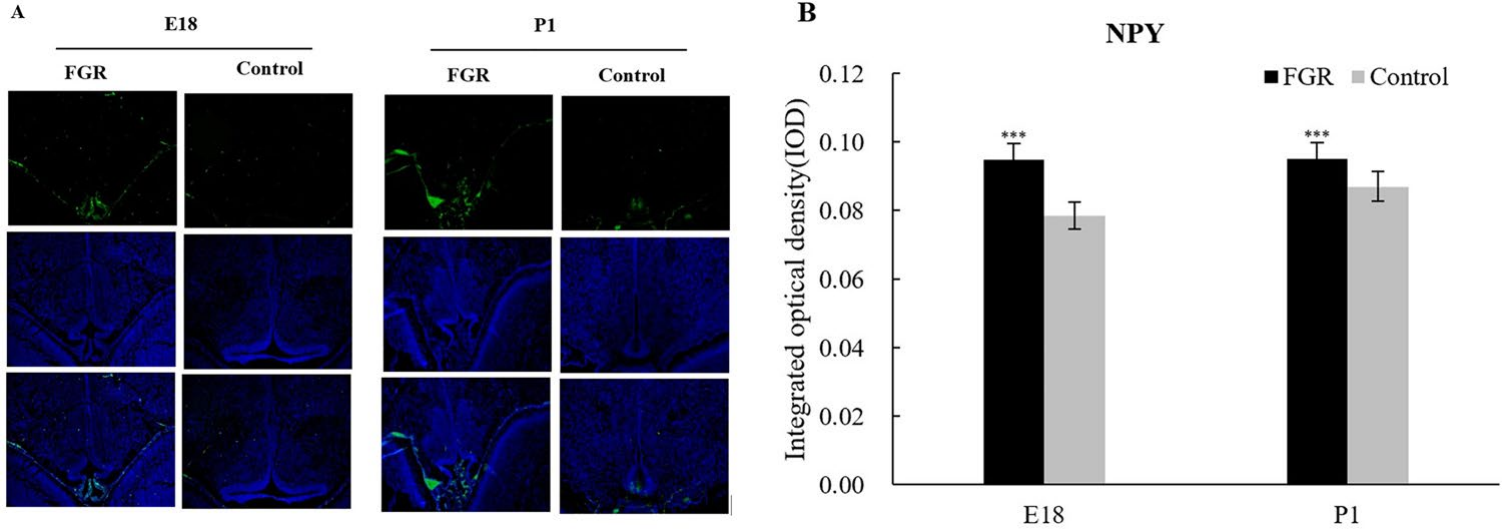
NPY expression in FGR and control group measured by immunofluorescence staining (100 ×). The green fluorescence is NPY expression, and the blue fluorescence is DAPI nuclear staining. The third line is the fused image. E18: embryonic day 18 cohort; P1: postnatal day 1 cohort. ***P < 0.001

We used IF to detect NPY expression qualitatively and positionally. As is shown in Fig. 1, green fluorescence is the expression part of NPY; Blue fluorescence is the nuclear expression part. NPY was mainly distributed around the third ventricle. Compared with the control group, the fluorescence intensity of NPY in the FGR group was brighter (Fig. 1A). The result of semi-quantitative analysis also showed that there was a higher value of integrated optical density (IOD) in the FGR group than that in the control group (Fig. 1B).

### The protein and mRNA expression of DNMT1 decreased in the FGR group, while that of Bsx and NPY increased

**Fig. 2:**
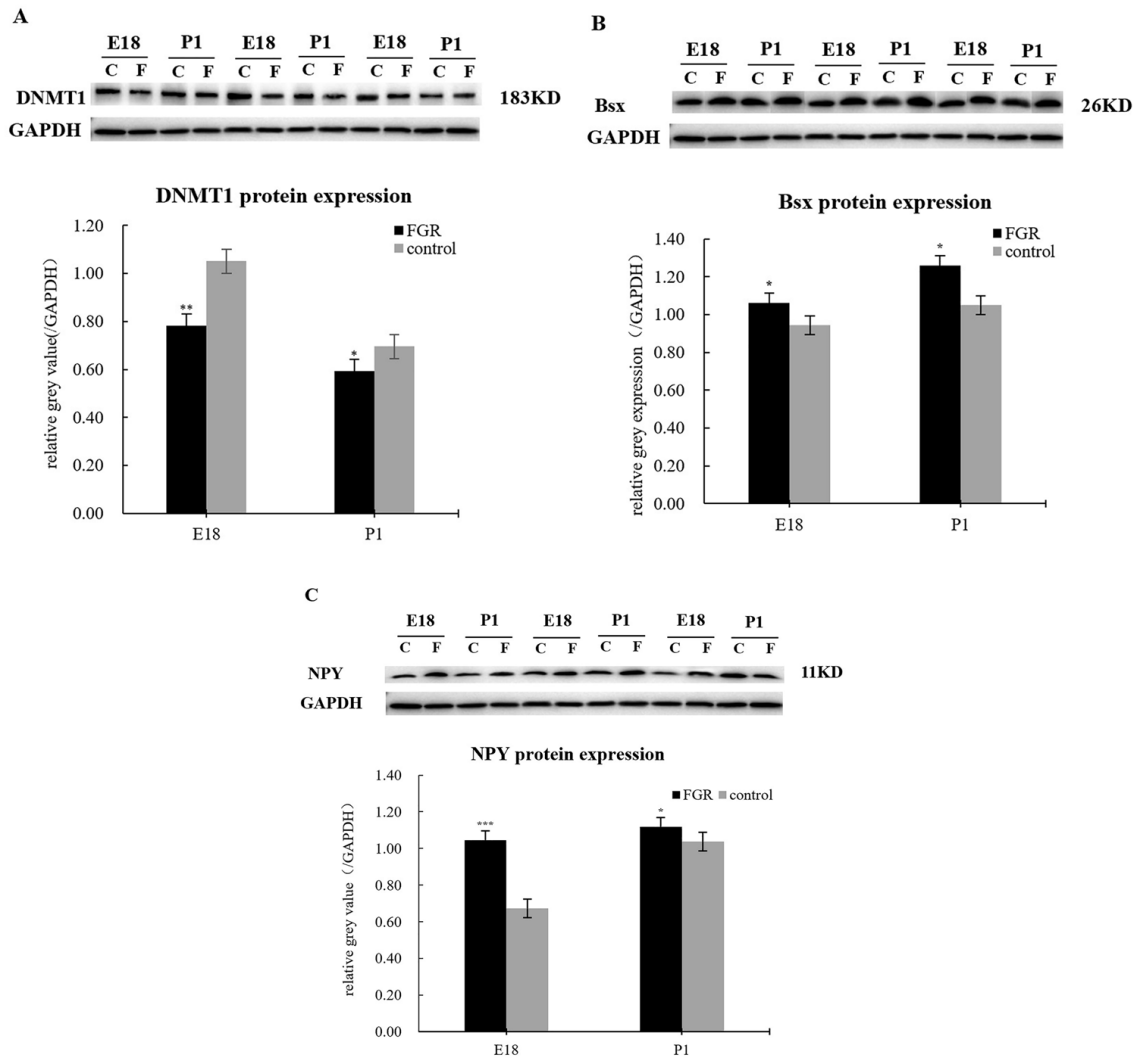
Protein expression of DNMT1 (**A**), Bsx (**A**) and NPY (**C**) of female rat hypothalamus in the FGR and control group. C: the control group; F: the FGR group; E18: embryonic day 18 cohort; P1: postnatal day 1 cohort. GAPDH: endogenous reference. *P < 0.05; ***P < 0.001

**Fig. 3:**
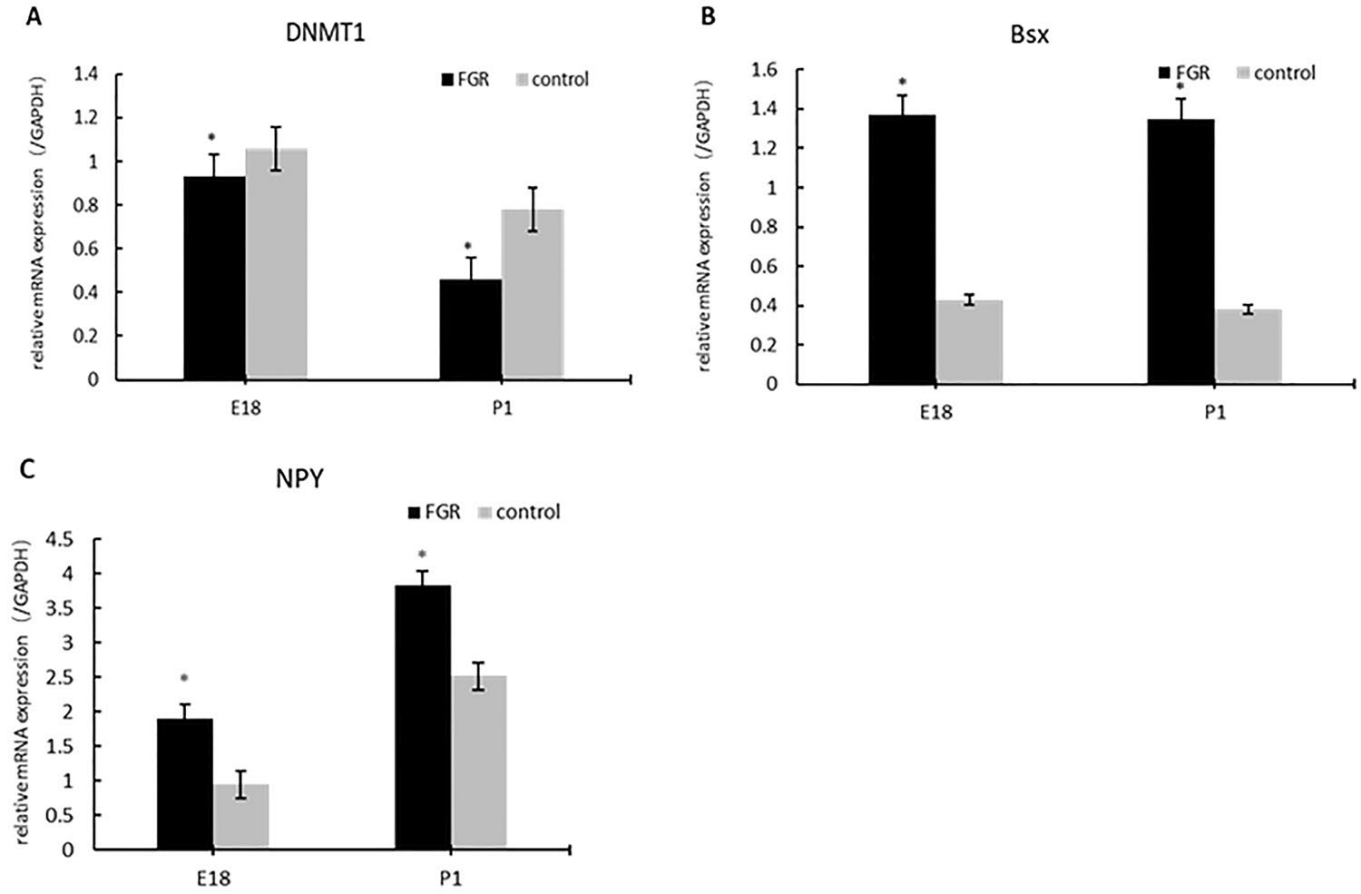
The mRNA expression of DNMT1 (**A**), Bsx (**B**) and NPY (**C**) of rat hypothalamus in FGR and control group. E18: embryonic day 18 cohort; P1: postnatal day 1 cohort. GAPDH: endogenous reference. *The difference was statistically significant (*P* < 0.05)

Hypothalamus tissues were used to detect the expression of DNMT1, Bsx, and NPY by RT-qPCR and WB. Results are shown in Figs. 2 and 3. Compared with the control group, the DNMT1 protein expression in the FGR group decreased. both in the E18 and P1 cohort (Fig. 2A). On the contrary, the Bsx and NPY protein expression increased (Fig. 2B, C). Besides, the result also displayed a decreased trend of DNMT1 protein expression from E18 to P1, while Bsx and NPY exhibited the opposite trend.

The DNMT1 mRNA expression in the FGR group, either the E18 cohort or the P1 cohort, was significantly lower than that in the control group (*P* < 0.05). The difference was much apparent in the E18 cohort (Fig. 3A). The Bsx and NPY mRNA expression in the FGR group, both the E18 cohort and P1 cohort, were significantly higher than that in the control group (Fig. 3B and C) (*P* < 0.05). Besides, the result also suggested a decreased trend of DNMT1 mRNA expression from E18 to P1, while NPY exhibited the opposite trend.

### The methylation level of the Bsx promoter in the hypothalamus decreased in the FGR group

**Fig. 4:**
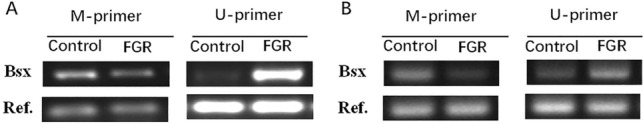
Methylation level of Bsx promoter region in rat hypothalamus of FGR and control group. **A**: the result of the E18 cohort. **B**: the result of the P1 cohort. E18: embryonic day th; P1:postnatal day 1. Ref.: endogenous reference. M-primer: Methylation primer. U-primer: un-methylation primer

To clarify whether there is a methylation change on Bsx, we detected the methylation level of Bsx by MSP. The result is shown in Fig. 4. The methylation level of Bsx promoter region in FGR group was lower than that in the control group, while the un-methylation level was higher (Fig. 4).

### The expression of Bsx and NPY increased in NPCs cultured in vitro with DNMT1-specific inhibitor

To clarify whether DNMT1 can regulate the expression of Bsx and NPY, we cultured NPCs in a differentiation medium with or without DNMT1-specific inhibitor. 5-AZA, a kind of DNMT1-specific enzyme activity inhibitor, was used in this experiment.

**Fig. 5:**
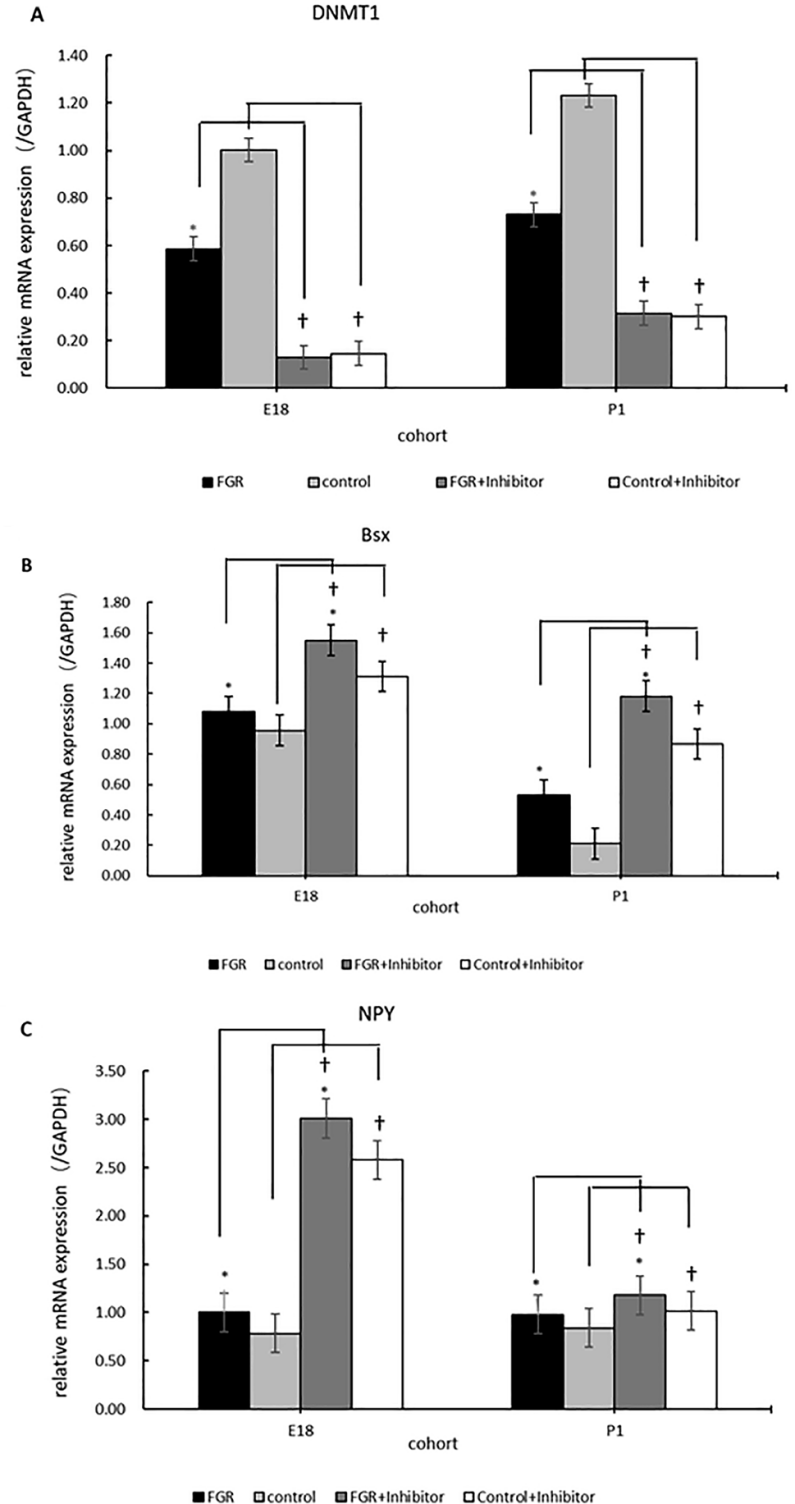
The DNMT1 (**A**), Bsx (**B**) and NPY (**C**) mRNA expression in hypothalamic NPCs cultured in vitro with or without 5-AZA. 5-AZA: DNMT1-specific enzyme activity inhibitor (20μ M). E18: embryonic day 18 cohort; P1: postnatal day 1 cohort. *The difference was statistically significant (*P* < 0.05) between FGR and the control group. ^†^The difference was statistically significant (*P* < 0.05) between groups cultured with or without 5-AZA

As shown in Fig. 5A, the DNMT1 mRNA expression in the FGR group was lower than in the control group (*P* < 0.05). In the groups that NPCs were cultured in vitro with 5-AZA, the expression of DNMT1 was inhibited (*P* < 0.05). There was no significant difference between groups cultured with 5-AZA.

In Fig. 5B, compared with the control group, the mRNA expression of Bsx in the FGR group increased with statistical significance (*P* < 0.05). The Bsx expression in groups cultured with 5-AZA was much higher than that cultured without 5-AZA (*P* < 0.05). NPY displayed the same expression mode as Bsx (Fig. 5C).

### The methylation level in the Bsx promotor region decreased in NPCs cultured in vitro with DNMT1-specific inhibitor

**Fig. 6:**
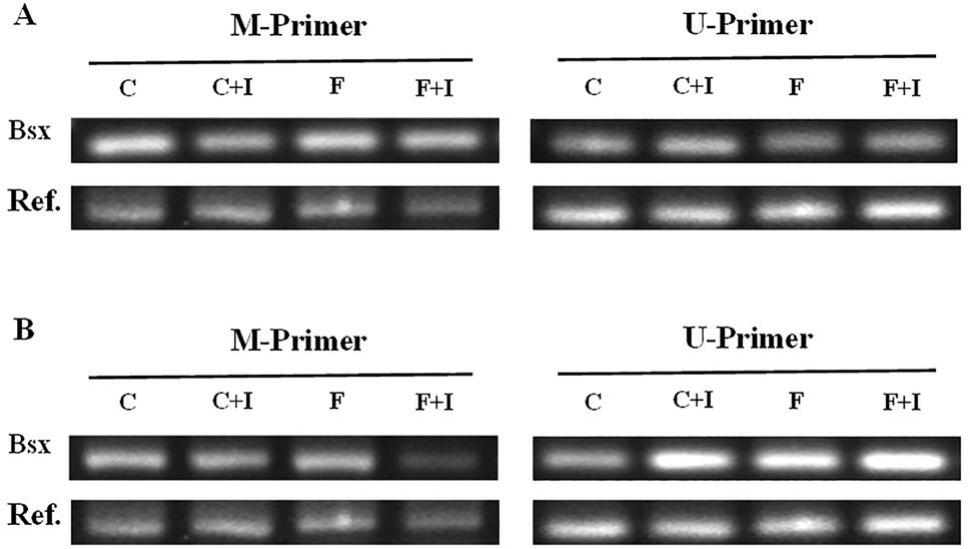
Methylation level in Bsx promoter region in hypothalamic NPCs cultured in vitro with or without 5-AZA. 5-AZA: DNMT1-specific enzyme activity inhibitor (20μ M). **A** the result of the E18 cohort. **B** the result of the P1 cohort. E18: embryonic day 18; P1:postnatal day 1. Ref.: endogenous reference. M-primer: Methylation primer. U-primer: un-methylation primer. **C** NPCs from control group cultured without DNMT1 inhibitor; C + I: NPCs from control group cultured with DNMT1 inhibitor; **F** NPCs from FGR group cultured without DNMT1 inhibitor; F + I: NPCs from FGR group cultured with DNMT1 inhibitor

We also tested Bsx methylation levels among groups. The result is shown in Fig. 6. Compared with the control group, the methylation level in the Bsx promoter region in the FGR group decreased. Meanwhile, the methylation level decreased in NPCs cultured in vitro with 5-AZA compared with those cultured without the inhibitor. The inhibit phenomenon was much more evident in the P1 cohort than in the E18 cohort (Fig. 6).

## Discussion

Epigenetic mechanisms are emerging as mediators linking intrauterine environmental exposures with programmed alterations in gene expression that impact offspring growth and development [20]. In this work, we first identified that DNMT1 mediates the expression of Bsx and NPY by influencing methylation levels in the Bsx promoter region in early embryonic neurogenesis, suggesting that the changes of early undernutrition on offspring DNA methylation preset and programmed obesity and metabolic syndrome.

Currently, there are many methods to establish the FGR rat model, such as 50% energy restriction, uterine artery ligation, pharmacological method, chronic nitric oxide synthase inhibition-induced method, and so on [21–23]. The 50% diet restriction method is widely used, but maternal rats are prone to mania in the state of hunger. The incidence of abortion, stillbirth, and postpartum feeding would increase. Yue Jing et al. adopted an 8% low-protein diet combined with 75% diet restriction modeling and achieved an excellent modeling success rate. In this experiment, we successfully established the FGR rat model by 75% energy restriction combined with 8% low-protein fed. The average success rate of modeling was 83.5%.

So far, it is well established that FGR and small birth weight lead to the initial increase in type 2 diabetes mellitus (T2DM), obesity, hypertension, dyslipidemia, insulin resistance and series of sequelae [24]. The association of FGR and long-term obesity remains an intersectional difficulty in both perinatal and endocrine medicine. It must be emphasized that poor growth in utero can modify neuroendocrine and metabolic systems. In particular, alterations in fetal adipose tissue development, hormone statuses as well as epigenome lead to later life obesity following intrauterine growth restriction [25–29]. Here, we highlight the potential linkages underlying relationship between FGR and altered chromatin structure.

Actually, hypothalamic appetite regulatory systems develop and mature in utero and early infancy [30]. There were abundant data indicating that intrauterine growth restriction exposure was associated with increased NPY levels [31–34]. Based on these, in this study, we detected high concentrations of NPY in the hypothalamus of FGR group. The result suggested abnormal differentiation exists in hypothalamic NPCs. Hypothalamus NPCs tend to differentiate into appetite-promoting neurons, resulting in hyperactivity of appetite-promoting activity and obesity.

Besides, since Bsx represents an essential factor for NPY/AgRP neuronal function [11], we conclude that Bsx may also be required for FGR-induced energy imbalance. Previous evidence indicated that, in Bsx mutant mice, NPY/AgRP expression as well as hypothalamic control of locomotory behavior depended on Bsx function [11]. In addition, Bsx has been shown to directly bind the promoter regions of Agrp and Npy [35]. In the present study, Bsx and NPY co-expressed much higher in the FGR hypothalamus than in the control group.

To further explore the mechanisms of FGR in altering orexigenic neuropeptides, we took DNA methylation (DNA methyltransferase; DNMT1) into consideration. Desai et al. confirmed that DNMT1 participants in the maintenance and steady-state regulation of hypothalamic NPCs proliferation to ensure the reserve of NPCs pool and the occurrence of a correct number of neurons [18]. Thus, we measured that the methylation level in Bsx promoter region in FGR group was lower than that in the control group, suggesting that the increased expression of Bsx may be related to the decreased methylation level in the Bsx promoter region. Combined with low expression of DNMT1, we suggested that DNMT1 might regulate Bsx expression through methylating specific gene sites of Bsx.

To further identify DNMT1 roles in Bsx and NPY abnormally high expression, we conducted an intervention test by using hypothalamic NPCs cultures in vitro. 5-AZA, a specific inhibitor of DNMT1 catalytic enzyme activity, was introduced to evaluate the mechanistic link between DNMT1 and Bsx. Notably, blockage of DNA methylation prevented the DNMT1 down-regulating action on Bsx and NPY mRNA expression. In other words, the expression of Bsx and NPY increased even further in the group cultured with 5-AZA. In addition, this effect was observed in both P1 and E18 cohort, suggesting that DNMT1 might play a continuous role in regulating Bsx and NPY expression either in-uterus or early postnatal stage. Of note, the differentiation of Bsx high expression between FGR- and control-groups with 5-AZA treated indicated a significant but non-unique role of DNMT1 in neural programming. Interestingly, using DNMT1-specific inhibitor, the concentration of NPY in the P1 cohort did not elevate as much as that in E18 cohort. The altered expression might be the consequence of the maturation in central nervous system occurring throughout from the intrauterine period to early years of life [36–38]. On the other hand, we observed that the expression of NYP did not exhibit a coordinate increase as Bsx did, whereas the change ranges were consistent with the methylation status of Bsx promotor region in MSP test. However, as to why the methylation status of Bsx promotor region differs from prenatal to postnatal, the theories therein are not known. Given the fact that the offspring rats were not exposed to the same physiological conditions, and DNA methylation could be affected by metabolic phenotypes [39], it is tempting to speculate that gene expression variability due to DNA methylation may also be adjusted and steadied in preparation for postnatal life. Therefore, as nervous system continues to develop after birth in rodents [40], it is of importance for future researches to substantiate the long-term effects. Besides, further investigation is warranted to illustrate whether the experimental results are suitable for humans.

In conclusion, here, our data support the hypothesis that the onset of Bsx and NPY activity occurs at the early embryonic development stage. Moreover, DNMT1 may regulate Bsx expression through methylation, which further affects the normal development of the hypothalamic appetite regulation pathway. These findings provide insight into the mechanism that the change of methylation level in the Bsx promoter region mediated by DNMT1 may be one of the reasons for the abnormal development of NPY in FGR offspring. Obviously, maternal undernutrition can alter epigenetic marks and cause widespread consequences.

## Data Availability

The data presented in this study are available on request from the corresponding author.
